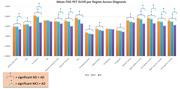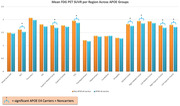# Relationship of Mito‐Nuclear Genetics to FDG‐PET Alzheimer’s Disease Biomarkers in Aging

**DOI:** 10.1002/alz.093820

**Published:** 2025-01-09

**Authors:** Robyn A Honea, Eric D Vidoni, Rebecca J Lepping, Jill K Morris, Heather M Wilkins, Jeffrey M Burns, Russell H Swerdlow

**Affiliations:** ^1^ University of Kansas Alzheimer’s Disease Research Center, Fairway, KS USA; ^2^ University of Kansas Medical Center, Kansas City, KS USA; ^3^ Hoglund Biomedical Imaging Center, University of Kansas Medical Center, Kansas City, KS USA

## Abstract

**Background:**

A growing amount of data has implicated the various roles of nuclear genes involved in mitochondrial function, and mitochondrial genes, on risk for Alzheimer’s disease (AD) and AD neuroimaging biomarkers. To date, no studies have investigated the relationship of mitochondrial haplogroups or the APOE and TOMM40 genes on brain glucose metabolism, a sensitive early marker of metabolic decline and possible mitochondrial dysfunction in AD.

**Method:**

We analyzed regional standard uptake value ratio (SUVR) differences in 18F‐fluorodeoxyglucose positron emission tomography (FDG‐PET) using SPM12 and CAT12 software between nondemented (ND, n = 69), mild cognitively impaired (MCI, n = 19) and AD (n = 18) groups in a sample of individuals from the University of Kansas Alzheimer’s Disease Research Center Cohort, controlling for age, sex, and education. We then tested whether genetic variation in APOE (e4‐carriers vs. non‐carriers), TOMM40 rs2075650 (‘650 G‐allele carriers vs. A‐Allele) or mitochondrial haplogroup significantly influenced regional differences in glucose metabolism in nondemented individuals, as they made up the bulk of our sample of individuals with these scans.

**Result:**

We found that, as expected, individuals with AD had significantly decreased glucose metabolism across all regions except the motor cortex and the cerebellum compared to ND individuals (Figure 1). Our results across various gene groups, controlling for age, sex, education, and with and without APOE e4 showed that there were no significant differences in FDG‐PET SUVR between ND TOMM40 ‘650 G‐allele carriers, or mitochondrial haplogroups (H, JT, and UK groups combined), however APOE e4‐carriers had significantly lower glucose metabolism in the anterior cingulate, superior medial frontal, posterior cingulate, medial frontal, right middle frontal, and bilateral superior frontal cortices than e4‐non carriers (Figure 2). There were no significant effects of APOE e4 on glucose metabolism in limbic regions.

**Conclusion:**

The APOE e4 allele may have more impact on FDG‐PET in default mode regions (posterior and anterior cingulate, medial frontal cortex) than TOMM40 '650, a larger study in cognitively healthy individuals will be needed to confirm these results.